# Progression of Parkinson's Disease Pathology Is Reproduced by Intragastric Administration of Rotenone in Mice

**DOI:** 10.1371/journal.pone.0008762

**Published:** 2010-01-19

**Authors:** Francisco Pan-Montojo, Oleg Anichtchik, Yanina Dening, Lilla Knels, Stefan Pursche, Roland Jung, Sandra Jackson, Gabriele Gille, Maria Grazia Spillantini, Heinz Reichmann, Richard H. W. Funk

**Affiliations:** 1 Institute of Anatomy, Medical Faculty Carl Gustav Carus, Dresden University of Technology, Dresden, Germany; 2 Department of Neurology, Medical Faculty Carl Gustav Carus, Dresden University of Technology, Dresden, Germany; 3 Center for Brain Repair, University of Cambridge, Cambridge, United Kingdom; 4 Department of Internal Medicine I, Medical Faculty Carl Gustav Carus, Dresden University of Technology, Dresden, Germany; 5 Experimental Center, Medical Faculty Carl Gustav Carus, Dresden University of Technology, Dresden, Germany; 6 International Max-Planck Research School, Max-Planck Institute for Cell Biology and Genetics, Dresden, Germany; Julius-Maximilians-Universität Würzburg, Germany

## Abstract

In patients with Parkinson's disease (PD), the associated pathology follows a characteristic pattern involving *inter alia* the enteric nervous system (ENS), the dorsal motor nucleus of the vagus (DMV), the intermediolateral nucleus of the spinal cord and the substantia nigra, providing the basis for the neuropathological staging of the disease. Here we report that intragastrically administered rotenone, a commonly used pesticide that inhibits Complex I of the mitochondrial respiratory chain, is able to reproduce PD pathological staging as found in patients. Our results show that low doses of chronically and intragastrically administered rotenone induce alpha-synuclein accumulation in all the above-mentioned nervous system structures of wild-type mice. Moreover, we also observed inflammation and alpha-synuclein phosphorylation in the ENS and DMV. HPLC analysis showed no rotenone levels in the systemic blood or the central nervous system (detection limit [rotenone]<20 nM) and mitochondrial Complex I measurements showed no systemic Complex I inhibition after 1.5 months of treatment. These alterations are sequential, appearing only in synaptically connected nervous structures, treatment time-dependent and accompanied by inflammatory signs and motor dysfunctions. These results strongly suggest that the local effect of pesticides on the ENS might be sufficient to induce PD-like progression and to reproduce the neuroanatomical and neurochemical features of PD staging. It provides new insight into how environmental factors could trigger PD and suggests a transsynaptic mechanism by which PD might spread throughout the central nervous system.

## Introduction

Parkinson's Disease (PD) is a highly prevalent disease affecting 0.3% of the general population and 1–3% of the population over the age of 65 [1]. PD is slowly progressive, and is characterized by dysfunction of the somatomotor system (i.e., rigidity, bradykinesia, postural instability, gait dysfunction and tremor), which usually dominates the clinical picture of sporadic PD [2]. The main symptoms are caused by the progressive degeneration of the nigrostriatal dopaminergic pathway [3], [4] These complaints, however, are often preceded or accompanied by other symptoms that also emerge during the disease course [5], [6], [7]. Hyposmia and gastrointestinal alterations are among the non-motor signs that develop early, often preceding motor symptoms by years [8], [9], [10], [11], and are normally accompanied by autonomic dysfunction [12], [13] as well as the experience of pain [14], [15].

A pathological hallmark of PD is the accumulation of filamentous, cytoplasmic inclusions consisting mainly of alpha-synuclein aggregations in the form of Lewy bodies (LB) or Lewy neurites (LN) [16], [17]. They are found in certain areas of the central nervous system (CNS), e.g. the dorsal motor nucleus of the vagus (DMV), the intermediolateral nucleus in the spinal cord (IML), the locus coeruleus (LC) and the olfactory bulb (OB), and of the peripheral nervous system (PNS) e.g., celiac ganglia and ENS, of PD patients [18], [19], [20], [21]. These pathological findings are usually associated with an inflammatory response [22] and phosphorylation of the alpha-synuclein (Ser129) which accumulates as a component of LB in the brains of patients with alpha-synucleinopathies [23].

Although genetic mutations contribute to the development of rare familial forms of PD (e.g. mutations in alpha-synuclein, Parkin, LRRK2, PINK1 genes) [24], [25], [26] most of the cases are sporadic and due to unclear aetiologies. It has been postulated that mitochondrial dysfunction [27], oxidative stress [28], inflammatory response [29] and protein mishandling may play an important role in the pathogenesis of sporadic PD [30]. On the other hand, multiple epidemiological studies suggest an association between pesticides and the incidence of PD [31].

Many studies have used different pesticides and routes of administration in order to reproduce pathological and clinical findings of PD in animals [32]. These models show different combinations of the clinical and pathological features of PD, such as the selective loss of TH-positive neurons, the presence of LB in the SN, impaired striatal dopaminergic innervation or motor dysfunction. Nevertheless, they fail to reproduce the complete spectrum of pathological disease progression and provide little information on the risk of environmental exposure because these methods bypass the physical and metabolic defences of the organism. Moreover, systemic administration of rotenone in C57BL/6J mice has failed to reproduce any of the pathological features of PD [33].

Braak and colleagues have suggested that the pathological process starts in the ENS and OB progressing into the CNS through anatomically connected structures [21]. Interestingly, it has been shown that alpha-synuclein oligomeres can be endocytosed by neurons and induce alpha-synuclein aggregation in primary neuronal cultures [34], [35]. Moreover, Desplats and colleagues have shown inclusion formation and neuronal cell death through neuron-to-neuron transport of alpha-synuclein both *in vivo* and *in vitro* [36]. In our study we wanted to investigate whether the local effect of ingested pesticides (orally administered) on the intestinal track could induce alpha-synuclein accumulation in the ENS and thereby induce PD-like pathological progression predicted by Braak's model.

## Results

In order to test whether the local effect on the ENS of an orally administered pesticide could reproduce the progression of PD predicted by Braak's model, we decided to administer rotenone intragastrically to one-year old mice using a gastric tube.

### Method Validation

To determine that rotenone did not reach the systemic blood or the CNS, we performed HPLC analysis on blood, brain and brainstem samples of non-experimental C57BL/6J mice exposed to rotenone before starting the experiments. Blood samples were extracted from 8 week-old mice treated with 2.5, 5, 10 and 20 mg/kg rotenone for 7 days. Brain and brainstem samples were obtained from one-year old mice treated with 5, 10 and 20 mg/kg rotenone for 7 days. We were unable to detect rotenone via HPLC in the blood at doses of 5 mg/kg or lower and in the brainstem or brain at doses of 10 mg/kg or lower (Figure S1). Therefore, we decided to use a dose of 5 mg/kg rotenone to treat experimental mice. HPLC analysis made on weeks 2, 4 and 10 of treatment showed the same results (data not shown).

Rotenone is an extremely potent mitochondrial Complex I inhibitor. In order to further confirm the systemic absence of rotenone, we decided to measure mitochondrial Complex I activity in brain and muscle samples. The results showed no mitochondrial Complex I activity reduction in muscle or brain of 1.5 months treated mice when compared to the controls (Figure S1).

### Rotarod Test

To investigate whether treated mice showed motor dysfunction, we performed an accelerating rotarod test, which provides a more discriminating way to correlate motor deficits against lesion size [37]. The results of the rotarod test, performed with an initial speed of 4 rpm and an acceleration of 0.3 rpm/sec, showed a significant decrease (p<0.05) in the rodent's ability to remain on the rod between 3 month treated mice and controls, but not between the performance of 1.5 month treated mice and controls (Figure S1). No difference was found between the treated groups and controls when an acceleration of 0.2 rpm/sec was used (data not shown).

We then examined whether there were alpha-synuclein PD-like alterations in ENS, IML, DMV or the SN and if these alterations were progressive.

### Effects of Rotenone on the ENS

**Figure 1:**
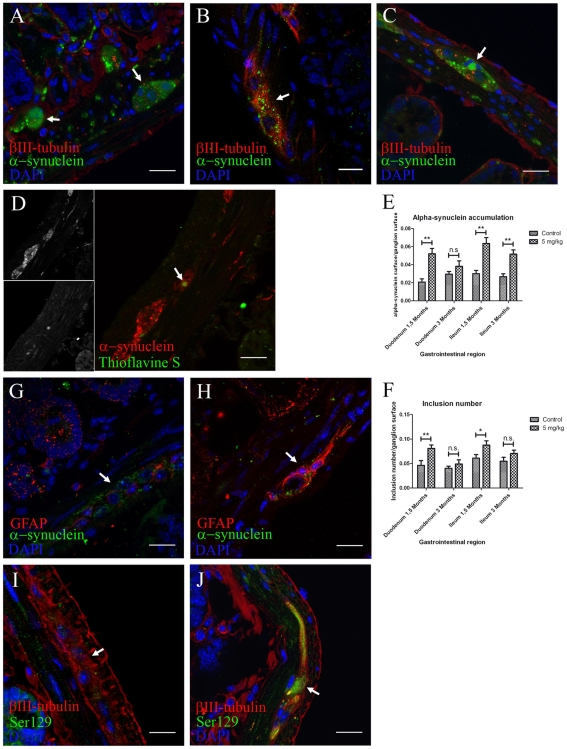
Locally administered rotenone induces alpha-synuclein phosphorylation, accumulation and aggregation with gliosis in ENS ganglia. (scale bars 20 µm). **A**, **B**, **C**, anti βIII-tubulin, alpha-synuclein and DAPI staining in duodenum (**B**) and ileum (**A**,**C**) sections. Arrow in **B**, 1.5 months treatment induced an increased alpha-synuclein punctate pattern inside enteric nervous system ganglia when compared to 3 months controls (**A**). Arrow in **C**, 3 months treatment induced formation of larger alpha-synuclein inclusions (|>6 µm). **D**, immunofluorescence staining using anti-alpha-synuclein, Thioflavine S and DAPI. Arrow in **D**, only 3 month treated mice showed aggregation of these larger alpha-synuclein accumulations. **E**, **F**, quantification of the experiment shown in **A**–**C** was made using automatic segmentation and entropy-based thresholding methods. Single-asterisk, P<0.05, and double-asterisk, P<0.01. **E**, each column represents total alpha-synuclein surface/ganglion surface. **F**, each column represents total number of alpha-synuclein inclusions/ganglion surface. All graphs show mean ± s.e.m. **G**, **H**, max-projection of staining against GFAP, alpha-synuclein and DAPI on duodenum sections from control (**G**) and treated (**H**) mice. **I**, **J**, max-projection of anti-βIII-tubulin, anti-phospho-alpha-synuclein (Ser 129) and DAPI staining on duodenum sections from control (**I**) and treated (**J**) animals.

We detected alpha-synuclein in ENS neurons from the duodenum and ileum of both control and treated animals. However, the distribution, inclusion number and amount of alpha-synuclein differed between treated and control animals. Control mice showed alpha-synuclein mainly in the periphery of the neuronal soma (Figure 1A) while in treated mice, the amount of alpha-synuclein was increased and it was present both inside and outside the neural soma (Figure 1B), with larger alpha-synuclein accumulations (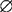
>6 µm) in 20% of the analyzed ganglia after 3 months treatment (Figure 1C). To investigate whether alpha-synuclein was aggregated, we double stained with alpha-synuclein and Thioflavine-S, a marker for β-sheet formation within filamentous aggregates. We observed alpha-synuclein aggregation only in the larger accumulations identified in mice treated for 3 months (Figure 1D). Associated alpha-synuclein phosphorylation and gliosis was confirmed by the presence of GFAP and Ser-129 phosphorylated alpha-synuclein in ENS ganglia from treated (Fig. 1H and 1J) but not control mice (Figure 1G and 1I). Immunohistochemical staining for alpha-synuclein using an alternative alpha-synuclein antibody (Syn-1) confirmed the presence of synuclein staining within ganglia of ENS. No specific staining with AT8 antibody (phosphorylated tau marker) was detected and control immunostaining using only secondary antibodies showed no unspecific staining.

Image analysis using Image J Software showed that the total surface stained with alpha-synuclein inside the ganglia and the number of inclusions significantly increased in the duodenum and ileum after 1.5 months rotenone treatment when compared to controls (p<0.01) (Figure 1E and 1F). Interestingly, the value of these parameters decreased after 3 months treatment coinciding with the appearance of larger inclusions of aggregated alpha-synuclein (Figure 1C).

### The Local Effect of Rotenone on the ENS Leads to Alpha-Synuclein Accumulation in the IML and the DMV

**Figure 2:**
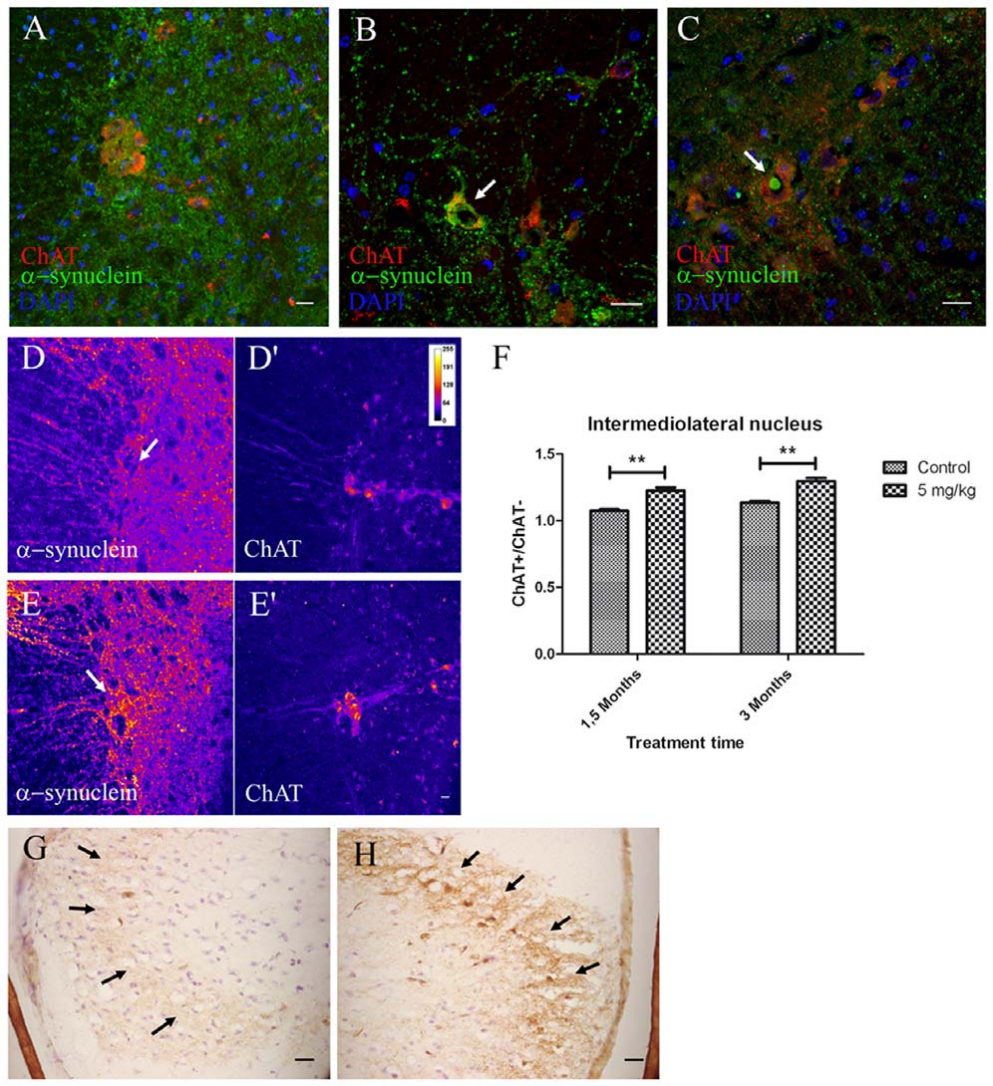
Intracellular and axonal alpha-synuclein increases in the intermediolateral nucleus and the dorsal horn lamina I layer of the spinal cord after oral rotenone treatment. (scale bars 20 µm) **A**, **B**, **C**, Immunostaining against alpha-synuclein and choline acetyl transferase (ChAT) in spinal cord sections showing the intermediolateral nucleus ChAT^+^ neurons from 3 months control mice (**A**), 1.5 months (**B**) and 3 months (**C**) treated mice. Arrow in **B**, colocalization of increased intracellular alpha-synuclein and ChAT^+^ stainings in the IML. Arrow in **C**, large alpha-synuclein inclusion (|>7.5 µm) inside an IML ChAT^+^ neuron. **D**–**E**, fluorescence intensity color-coded images from 3 months control (**D**, **D'**) and 3 months treated mice (**E**, **E'**) spinal cord sections stained using DAPI and alpha-synuclein and ChAT antibodies. Arrows in **D** and **E**, areas in the proximity of ChAT^+^ neurons. **F**, mean fluorescence quantification of experiment shown in **D** and **E**. Double asterisk, P<0,01. Columns represent mean alpha-synuclein fluorescence in and around ChAT^+^ neurons in the IML/mean alpha-synuclein fluorescence in the region anterior to the IML. Graph shows mean ± s.e.m.. **G**, **H**, DAB-staining against apha-synuclein using synuclein-1 antibody in the dorsal horn of the spinal cord from 3 months treated (**H**) and control (**G**) mice. Arrows in **G**–**H**, lamina I layer of the dorsal horn.

Next, we wanted to determine whether the local effect of rotenone on the ENS could lead to alterations in the synaptically connected autonomic nervous system centers in the spinal cord and the brainstem (the IML and the DMV). Indeed, PD related pathological changes were detected in both sites. We observed an increase in alpha-synuclein and some large alpha-synuclein intracellular inclusions (2% of the analyzed images) in ChAT^+^ neurons of IML (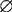
>7.5 µm) (Figure 2A–C) as well as increased alpha-synuclein staining in the dorsal horn layer I after 1.5 and 3 months treatment (Figure 2G and 2H) when compared to controls.

In order to test whether there was a correlation between alpha-synuclein pathology and cell death in IML neurons, we analyzed the ChAT^+^ neuronal population in the IML. Our results showed that even after 3 months of treatment there was no significant difference in the mean number of IML cholinergic neurons per section between treated (3.839 ± 0.2196, n = 5) and control (3.607 ± 0.2065, n = 5) mice.

We used image analysis to quantify alpha-synuclein increase by measuring fluorescence in the IML. Fluorescence measurements showed that the IML/IML adjacent region ratio of alpha-synuclein mean fluorescence intensity was increased in both 1.5 month (p<0.01) and 3 month-treated mice (p<0.01) when compared to controls (Figure 2D–F), thus suggesting an increase in alpha-synuclein in preganglionic sympathetic neuron processes. This difference was not observed in ChAT^+^ motor neurons of the anterior horn (Figure S2). These results were confirmed by immunohistochemistry on DAB stained sections, where the intensity of alpha-synuclein staining was greater in tissue sections from 3 month treated animals when compared to control sections (Figure S2). There were no differences in GFAP staining between treated and control mice in this area (data not shown).

**Figure 3:**
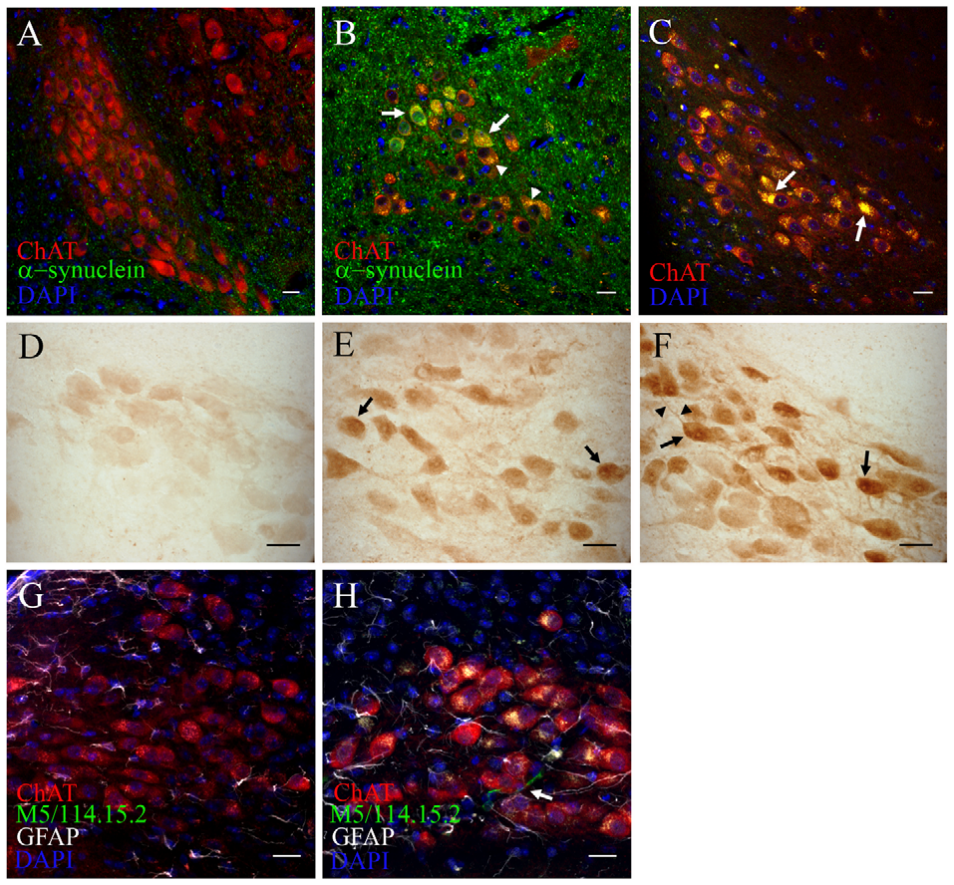
Intragastrically administered rotenone induces alpha-synuclein accumulation, oxidative stress and inflammation in the dorsal motor nucleus vagus. (scale bars 20 µm). **A**, **B**, double-immunofluorescence staining against alpha-synuclein and ChAT on DMV sections from 1.5 months control (**A**) and 1.5 months treated (**B**) mice. Arrows in **B**, increased intracellular alpha-synuclein in DMV neurons already after 1.5 months. Arrowheads in **B**, autofluorescent punctate inclusion pattern inside ChAT^+^ neurons. **C**, DMV sections stained with ChAT and DAPI were sequentially excited with 488 and 561 laser wavelengths. Arrows in **C**, large intracellular auto-fluorescent inclusions inside ChAT^+^ neurons of the DMV (arrows). **D**, **E**, **F**, Light microscopy images of alpha-synuclein staining from 1.5 months control (**D**), 1.5 months (**E**) and 3 months (**F**) treated mice. Arrows in **E** and **F**, increased staining intensity inside DMV neuronal soma in treated mice. Arrowheads in **F**, increased alpha-synuclein staining inside neuronal processes **G**, **H**, average-projection of triple-immunofluorescence staining against ChAT, GFAP, MHC II (clone M5/114.15.2) and DAPI on sections from control (**G**) and treated (**H**) mice after 3 month treatment. Arrow in **H**, activated microglial cell in the DMV.

In the DMV, 1.5 and 3 months rotenone treatment resulted in increased intracellular alpha-synuclein accumulation inside ChAT^+^ neurons (Figure 3B) when compared to controls (Figure 3A). We also observed an inflammatory associated reaction, with increased GFAP and some activated microglial cells around neurons of the DMV in treated but not control mice (Figure 3G and 3H). Remarkably, ChAT^+^ neurons from adjacent hypoglossus nuclei showed neither alpha-synuclein accumulations nor GFAP increase (data not shown). Control immunostaining with secondary antibodies showed auto-fluorescent inclusions inside DMV neurons from treated mice only (Figure 3C). Previous studies have identified auto-fluorescent inclusions as lipofuscin accumulations [38]. Lipofuscin granules mainly contain lipids. Therefore we used Sudan IV staining and confirmed them as lipofuscin inclusions (data not shown). In order to avoid auto-fluorescence interference, we also performed immunohistochemistry with DAB detection of alpha-synuclein on 1.5 and 3 months treated mice. This technique also showed alpha-synuclein accumulation in DMV neurons when compared to controls (Figure 3D–F). Alpha-synuclein was detected in both, neurons and the neuropil, however only in treated animals the staining of neural processes was visible (Figure 3F).

Finally, we analyzed the effect of rotenone treatment on the ChAT^+^ neuronal population in the DMV. Again, even after 3 months treatment no significant difference in the total number of cholinergic neurons between treated (1932 ± 294.7, n = 5) and control (1632 ± 281.3, n = 5) mice was detected.

Altogether, these results suggest that both directly (DMV) and indirectly (IML) with the ENS synaptically connected structures harbour accumulated alpha-synuclein after rotenone administration. However, accumulation of alpha-synuclein in these areas does not affect neuronal population in terms of cell loss.

### Alpha-Synuclein Pathology Progresses into the SN after Three Months Oral Rotenone Treatment

**Figure 4:**
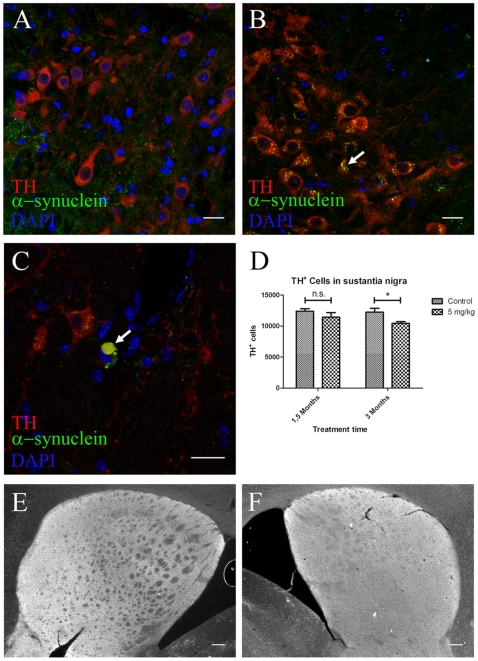
Alpha-synuclein accumulation and neuronal loss in the SNc after 3 but not 1.5 months intragastrical rotenone treatment. (**A**–**C**, scale bars 20 µm; **E**–**F**, scale bars 200 µm). **A**, **B**, **C**, immunostaining against TH, alpha-synuclein and DAPI on SNc sections from 1.5 months control (**A**) and 3 months (**B**–**C**) treated mice. Arrow in **B**, alpha-synuclein small inclusions inside TH^+^ neurons. Arrow in **C**, large alpha-synuclein inclusion (|>8.14 µm) inside a dopamineric neuron in the SN. **D**, stereological quantification (n = 3) of TH^+^ neurons in the SN from control and treated mice. Asterisk, P<0.05. Number of neurons was determined based on the optical fractionator principle using StereoInvestigator software (MicroBrightField Inc., Williston, USA). Each column represents total number of TH^+^ neurons in the SN in 1.5 and 3 months control and treated mice. Graph shows mean ± s.e.m. **E**, **F**, TH immunostaining on striatum in 1.5 months control (**E**) and 3 months treated (**F**) mice.

In order to investigate whether other PD-related CNS structures were affected, we analyzed the SN and the striatum of rotenone-treated and control mice. In the SN, we observed an increase in intracellular alpha-synuclein (Figure 4B) together with larger alpha-synuclein accumulations in the substantia nigra *pars compacta* (SNc) (Figure 4c) similarly to that within DMV and IML. Remarkably we also observed a significant 15.4% decrease (p<0.05) in the number of TH^+^ neurons (Figure 4D) in 3 month but not 1.5 month treated mice when compared to controls (Figure 4A).

The decrease in dopaminergic neurons could also be explained by a down regulation of the dopamine rate-limiting synthesizing enzyme tyrosine hydroxylase (TH). Therefore, we also performed a double-staining using DAB detection of TH together with Nissl's staining to quantify the total number of neurons in this area after three-month treatment in both groups (Figure S3A and S3B). We observed an increase in the amount of SNc cells in both groups (Figure S3C). This increase was significantly higher in treated mice than in control mice (Figure S3D). Interestingly, we also observed that the decrease in Nissl^+^ neurons between 3-month control and treated mice was 19% smaller than the decrease in TH^+^ neurons (Figure S3E). Altogether, these results suggest that together with a loss of TH^+^ neurons there are some dopaminergic neurons that might indeed have a lower TH expression and could, therefore, been missed in the stereological analysis based only on TH^+^ neurons.

We did not detect significant differences in the dopaminergic innervation of the striatum (Figure 4E–F), brain tau protein, lipofucsin granules or alpha-synuclein accumulation in other brain areas (e.g. cerebellum, cortex or striatum) (data not shown) between treated and control animals at any time-point, confirming that observed changes in alpha-synuclein accumulation and aggregation are confined within selected pathways of the intestine-cerebral axis.

## Discussion

It has been proposed that the PD-related inclusion body pathology in the nervous system progresses in six stages, starting at the OB and the ENS and affecting closely connected neural sites [39], [40]. Interestingly, the OB and the ENS are the only nervous system structures directly exposed to environmental substances.

Recently developed PD animal toxic models fail to reproduce the entire pathological spectrum of the disease. Moreover, they also avoid the important physiological defence mechanisms to external substances [32], [41], [42].

Actual reports concerning patients with PD who received transplants of embryonic mesencephalic neurons show that in some cases, mesencephalic neurons grafted into the striatum of PD patients develop both alpha-synuclein and ubiquitin positive LB [43], [44], [45]. According to these and other evidence [46], Brundin and colleagues proposed inflammation, oxidative stress, excitotoxicity, prion-disease-like mechanisms and loss of neurotrophic support as possible underlying mechanisms for PD propagation [47]. Interestingly, Desplats and colleagues have recently shown inclusion formation and neuronal cell death through neuron-to-neuron transport of alpha-synuclein both *in vivo* and *in vitro* [36].

Our results show that chronic treatment with intragastrically administered rotenone reproduces some of the reported PD pathological features – accumulation and aggregation of alpha-synuclein within ENS, DMV, IML and SN. These alterations occur without detectable levels of the pesticide in the blood or the brain and no inhibition of Complex I activity in muscle or brain even after 1.5 months of treatment. The HPLC method we employed was able to detect as little as 20 nM rotenone, but we were unable to detect rotenone in the blood with 5 mg/kg treatment, or in the CNS with 10 mg/kg treatment, see Results), which argues against systemic effect of rotenone being a causative factor for observed changes. Also, the results from other studies using animal models with higher systemic concentrations of rotenone [33], [48] and the absence of systemic Complex I inhibition speak against this possibility. Richter and colleagues have shown that subcutaneous administration of 2.5–4 mg/kg of rotenone for 30–40 days does not induce striatal degeneration or neuronal loss in the CNS of C57BL/6J mice. On the other hand, Betarbet and colleagues observed striatum degeneration before the SN was affected with the lowest dose (2.5 mg/kg) and treatment time (7 days) of systemically administered rotenone in rats, suggesting greater sensitivity of striatal nerve terminals. Remarkably, they did not observe degeneration anywhere else in the CNS.

We observed alpha-synuclein accumulation, phosphorylation, and inflammatory signs and loss of nigral TH^+^ neurons in our treated mice after 3 months of the treatment. Moreover, we also observed intracellular auto-fluorescent Sudan-IV^+^ lipofuscin inclusions. Lipofuscin has been considered a biomarker for oxidative stress and its presence may suggest an increase in oxidative stress and the accumulation of advanced glycation end products [49]. The inflammation pattern observed indicates that inflammation might not be involved in the propagation of the alteration but is perhaps a consequence of neuronal damage or alpha-synuclein accumulation *in situ*. We have also detected specificity in the nervous structures affected and only neuronal subpopulations that have direct connections to ENS showed alterations while nearby areas remained unaffected. We did not observe any alterations in the OB, striatal TH or brain tau protein levels as reported in other rotenone models [48], [50], [51], suggesting greater selectivity of the neuronal damage in our model.

Interestingly, the accumulation and aggregation of alpha-synuclein in the DMV and the IML did not result in neuronal death. However, we did detect a selective loss of dopaminergic neurons in the SN after three but not 1.5 months of treatment. One other explanation for this loss of nigral dopaminergic neurons could be the down regulation of TH expression in the dopaminergic cells. Therefore, we also performed a Nissl staining. Our results show that, although there is also a significant decrease in the total number of Nissl+ cells in the SNc between treated and control mice, this difference is smaller than that between TH^+^ neurons. Thus, suggesting that there exists a percentage of dopaminergic neurons with decreased TH expression. These results indicate that there is an increased sensitivity of central dopaminergic neurons to the accumulation of intracellular alpha-synuclein when compared to DMV and IML neurons. However, there was a non-significant decrease in the number of neurons at the latter sites, indicating that longer treatment periods may lead to significant reductions in the neuronal population.

Rotenone treatment resulted in measurable changes in motor coordination, as detected by differences in the rotarod test performance. This difference was only observed after 3 months of treatment – at the same time that neuronal loss in the SN was detected. Moreover, there seems to be a correlation between the percentage of dopaminergic loss (between 12% and 15.4%) and the difference observed in the rotarod test (13.3%). Rotarod test's results in rotenone models of PD could be somewhat controversial. This controversy is based on the difficulty to separate the effect of dopamine loss in the CNS and the effect of systemic inhibition of mitochondrial Complex I activity on the motor functions and the spontaneous motor activity of the mice [33]. Precisely because we did not observe systemic Complex I activity inhibition in our model, we believe these concerns are not applicable to our model. Nevertheless, these results are intriguing, as it is very unlikely that neuronal loss in the sustantia nigra alone without a reduction in striatal dopamine could be responsible for these alterations. The accelerating protocol used has been described to be a more discriminative test to correlate motor deficits against lesion size, and might therefore be more sensitive to small alterations. Unfortunately, the small amount of tissue available did not allow us to measure dopamine levels in the striatum of treated mice.

Recently, it has been shown that systemic administration of rotenone in rats also induces alpha-synuclein inclusions in the ENS [52]. Thus, partially corroborating our results. However, this model fails to reproduce the progression of the disease as alterations in the ENS and the CNS occur simultaneously due to the systematic effect of rotenone, and they do not show DMV or IML alterations. Also, alpha-synuclein containing processes and neurons extensively innervate the ENS in rats [53], differing from the mouse.

Although further mechanistic studies are necessary (i.e. strategically-placed nervous system lesions), these results strongly support an enteric nervous system spread hypothesis. The selectivity of neuronal alterations and the appearance of alpha-synuclein accumulation in the SN only at the latest treatment time-point allows us to speculate with the possibility of a direct mechanism responsible for this remarkable pattern of progression. Although the precise mechanism remains unclear, recently published data suggest that alpha-synuclein could be transported between the cells in vitro and in vivo, inducing its aggregation in recipient cells [36].

To our knowledge, this is the first animal model capable of reproducing the progression of PD-like pathology after acting solely on the ENS. Therefore, we strongly believe that this new animal model of the disease provides a better tool for understanding the mechanisms underlying the pathophysiology of PD and may help to pin-point novel pathways for therapeutic intervention.

## Materials and Methods

### Animal Procedures

One-year old C57BL/6J mice (Janvier, France) were housed at room temperature under a 12-h light/dark cycle. Food and water was provided *ad libidum*. Mice were divided into four groups (n = 5) and treated 5 days a week for 1.5 and 3 months. A stomach tube was used to administer 0.01 ml/g animal weight of rotenone solution (0.625 mg/ml rotenone (Sigma-Aldrich, Germany), 4% carboxymethylcellulose (Sigma-Aldrich, Germany) and 1.25% chloroform (Carl Roth, Germany)) corresponding to a 5 mg/kg dose. Controls were treated only with the vehicle (4% carboxymethylcellulose and 1.25% chloroform). Blood extraction for High Performance Liquid Chromatography (HPLC) analysis was obtained from the retina plexus using a glass capillary under general anaesthesia (0.01 ml/g of Ketamin i.p.). CNS-tissue was obtained after cervical dislocation. The rotarod test was performed after 1.5 and 3 month treatment as already described by others [37]. Briefly, mice were place on a rotating rod with a initial speed of 4 rpm. The speed of rotation was gradually increased (0,3 rpm/sec) and the rodent's ability to remain on the rotating rod (time to the first fall) was recorded. The test was repeated three times a day on each animal over three consecutive days. All animal experiments were carried out in accordance with the National Institutes of Health Guide for the Care and Use of Laboratory Animals, and protocols were approved by the Saxonian Committee for Animal Research, Germany.

### Measurement of Rotenone Levels in Plasma and CNS from Mice

Blood from 8 week old control mice and 8 week old mice treated with 2.5, 5, 10 or 20 mg/kg rotenone for one week (n = 3) was extracted 1, 2 and 3 hours after rotenone administration and pooled in the same tube. Serum samples were obtained by centrifugation at 3000 rpm (Micro 12–24, Hettich Zentrifugen, Germany). Serum was analysed using a modified HPLC protocol as previously described [54]. Brain and brainstem from control, 5 (n = 3), 10 (n = 3) and 20 mg/kg (n = 1) rotenone 5 day-treated one-year old animals were collected 1 and 3 hours after the last rotenone administration and processed as already described by others [41]. The detection threshold was determined by adding different concentrations of rotenone to control plasma before extraction. The methods were linear over a concentration range from 10 to 500 ng/ml, the inter-assay precision expressed as coefficient of variation was <10%. The limit of detection was 10 ng/ml.

### Complex I Activity Measurements

Brain and muscle samples were homogenized in 0.5–1 ml of buffer (120 mM KCl, 20 mM HEPES, 5 mM MgCl2, 1 mM EGTA; pH 7.2) supplemented with 5 mg/ml BSA using a small ultraturrax. The extract was then centrifuged for 6 min at 3000 rpm and the supernatant recentrifuged under the same conditions. The final supernatant was exposed to three cycles of rapid freeze-thawing in liquid nitrogen and Complex I activity was measured as the rotenone sensitive NADH dehydrogenase activity as described previously [55]. Citrate synthase activity was also measured in the same homogenates [56]. Complex I activity was related to that of the mitochondrial marker citrate synthase.

### Tissue Preparation for Immunostaining

Mice were killed with an overdose of ketamine and perfused transcardially with 4% paraformaldehyde in 0.1 M phosphate buffer (pH 7.4). Brain, spinal cord and gastrointestinal tract were removed and kept in 4% PFA for 24 h. Tissues were transferred into 30% sucrose, where they remained until equilibrium. Brains were then frozen using a shock-freezing technique and stored at −80°C. Brain sections (40 µm) were transferred to a 96 wells plate filled with a cryoprotectant solution containing 25% ethylene glycol and 25% glycerin in 0.05 M phosphate and stored at −20°C until free-floating immunostaining was performed. Spinal cords were separated into their cervical, thoracic and lumbar parts. The thoracic portion was then divided in three equidistant portions. Portions of the duodenum and ileum were also removed from the gastrointestinal tract. Both different spinal cord regions and gut pieces were then separately and vertically immersed in Tissue-Tek® and stored at −80°C.

### Immunohistochemistry

Immunofluorescence was performed on 40 µm (brain and spinal cord) and 20 µm (gastrointestinal tract) paraformaldehyde fixed frozen sections. Brain sections were stained using a free-floating immunostaining technique whereas spinal cord and gut sections were stained on the slide. Non specific background staining from CNS sections was blocked overnight at 4°C in blocking solution (5% donkey serum (Jackson Immunoresearch Laboratories), 0.4% Triton-X-PBS), whereas gut sections were blocked for two hours at room temperature (RT) using 5% donkey serum in 0.25% Triton-X-PBS. Sections were then incubated with the primary antibodies for 24 hours at 4°C, washed in PBS, incubated for one hour at RT with the fluorescent secondary antibodies, washed in PBS again and mounted using Vectashield® mounting medium for fluorescence with DAPI (Vectro Laboratories, USA). The following polyclonal primary antibodies were used: goat anti-ChAT (1∶500, Chemicon, USA), sheep anti-TH (1∶1000, Peel freeze, Rogers, AR USA), rabbit anti-alpha-synuclein (1∶400, Santa Cruz, USA); chicken anti-GFAP (1∶1000, Abcam, UK) and rat anti-MHC II clone M5/114.15.2 (1∶200, BD Pharmigen, CA, USA), rabbit anti-phospho-alpha-synuclein (phospho S129) (1∶50, Abcam, UK), chicken anti-βIII-tubulin (1∶500, Novus Biologicals, USA). These were coupled with the following secondary antibodies: Alexa® 488 donkey anti-rabbit, Alexa® 488 donkey anti-goat, Alexa®488 donkey anti-rat, Alexa® 555 and Alexa®594 donkey anti-sheep and donkey anti-goat (all 1∶500 and from Invitrogen, USA) and 647 donkey anti-chicken (1∶200, Jackson Immunoresearch Laboratories, USA). DAB immunohistochemistry was done as reported before [57]. Mouse anti alpha-synuclein (BD Transduction Laboratores, San Jose, CA USA), rabbit anti-TH (Peel freeze, Rogers, AR USA) or AT8 (Pierce Biotechnology, Rockford, IL USA) primary antibodies were used at 1∶1,000. Counterstaining was performed using a Nissl's staining as already described by others [58].

### Imaging

Immunostained sections were analysed using a Leica Leitz DMRD bright field microscope and a Zeiss confocal microscope (LSM 510, Carl Zeiss, Jena). Z-stacks from immunofluorescent gut sections were obtained using an Apo-63X objective. A Z-stack was acquired for 7 consecutive ganglia per gut section/animal, with 1 µm spacing in the Z-direction. The optical resolution limit for these images was 0.13 µm in the XY direction and 1 µm in the Z direction. Single images of the IML from spinal cord sections were acquired using the same microscope equipped with an Apo-25X objective. The optical resolution limit for these images was 0.48 µm in the XY direction. No additional post-acquisition manipulations of digital images were done. Digital images were then processed using ImageJ free software (Rasband, W.S., ImageJ, U. S. National Institutes of Health, Bethesda, Maryland, USA, http://rsb.info.nih.gov/ij/, 1997–2005), where only cropping of the original image and minor adjustment of the brightness and contrast were performed.

### Image Analysis

Immunofluorescence image analysis was made using ImageJ and performed by a third independent researcher, unaware of images origin. Z-stacks from gut sections were used for quantifying the alpha-synuclein droplet size. For each stack, the ganglion region was selected and isolated. Automatic segmentation of alpha-synuclein droplets was made using an entropy-based thresholding method, and operations on binary images. The in-focus plane for alpha-synuclein droplets was then searched for, by identifying the plane where droplet total surface was maximal. This sole plane was used for further analysis. The total alpha-synuclein surface and inclusion number were measured and normalized by ganglion/neuron area for statistical analyses. IML images were used to compute a fluorescence ratio, by normalizing the alpha-synuclein signal in the IML region by that of adjacent areas.

### Stereological Procedures

Every sixth section was stained against TH or ChAT and alpha-synuclein and used for stereological analysis. The number of TH^+^/ChAT^+^ neurons in the SN and the DMV was estimated using the Optical Fractionator principle [59], [60] with StereoInvestigator software (MicroBrightField Inc., Williston, USA) on a Zeiss Axioplan microscope and using a 20X objective. Total TH^+^ neuron number (N) was calculated using the formula N 1/4 SQ– · (t/h) · (1/asf) · 1/ssf, where Q– 1/4 is the total number of cells counted, t 1/4 is the section thickness, h 1/4 is the height of optical dissector, asf 1/4 is the area of sampling fraction 1/4 a(frame)/a(x,y step) and ssf 1/4 is the section sampling fraction.

### Semi-Quantitative Neuronal Cell Count on the IML

Spinal cord sections from upper, middle and lower levels of the thoracic spinal cord were stained against ChAT and alpha-synuclein. The number of cholinergic neurons in the IML per section was then counted in 10 sections per level/animal (30 sections per animal) and means were compared.

### Statistical Analysis

All data was tested for significance using two-way ANOVA, significance being p≤0.05.

## Supporting Information

Figure S1 Motor dysfunction in rotenone treated mice without detection of rotenone in blood or CNS. A, standard (50 ng/ml) and chromatogram from brain samples of 20, 10 and 5 mg/kg treated mice. B and C, quantification of rotenone levels in blood (B) and CNS (C). B, blood levels 1, 2 and 3 hours after treatment. Mice were divided in three groups (n = 3) and treated with 2.5, 5, 10 and 20 mg/kg rotenone (n = 3), 300 µl of blood was extracted 1, 2 and 3 hours after rotenone administration and pooled together for HPLC analysis. C, mice were treated for one week once a day with 5 (n = 3), 10 (n = 3) and 20 (n = 1) mg/kg rotenone, brain and brainstem were extracted 1 and 2 hours after last administration and prepared for HPLC analysis. D, mitochondrial Complex I activity in muscle and brain samples of 1.5 month treated mice. E, F, acceleration rotarod protocols were performed after 1.5 (E) and 3 (F) months of treatment using 0.3 rpm/sec acceleration rates. * in F is P is <0.05 taking together values from the three days, values based on Student's t test. All error bars correspond to ± s.e.m.
(1.37 MB TIF)

Figure S2 Rotenone administered orally induces alpha-synuclein accumulation in the IML but not in the motor neurons from the anterior horn of the spinal cord. (scale bars 20 µm). A, B, DAB stained spinal cord sections counterstained with cresoyl violet from control (A) and treated mice (B). Arrows in A and B, IML region of the spinal cord. C, D, fluorescence intensity color-coded images of ChAT+ motor neurons of the anterior horn from control (C) and treated (D) mice. E, mean fluorescence quantification of experiment shown in C and D. Columns represent mean alpha-synuclein fluorescence in and around ChAT+ neurons in the anterior horn/mean alpha-synuclein fluorescence in the region posterior to the motor neurons. Graph shows mean ± s.e.m.
(3.67 MB TIF)

Figure S3 Rotenone treatment causes neuronal depletion and TH down-regulation in the SNc. (A, scale bar 80 µm, B, scale bar 20 µm) A, B, DAB against TH stained midbrain sections counterstained using Nissl's staining from 3 month treated mice. Arrowheads in B are Nissl+ but not TH+ neurons, whereas arrows in B are Nissl+−TH+ neurons. C, stereological quantification of total TH+ and total Nissl+ neurons in the SNc of 3 month control and treated mice. D, difference in the total number of cells between stereological quantification based on Nissl's and TH stainings in 3 month control and treated mice. E, neuronal decrease using DAB against TH (left column) and Nissl stainings (right column). * in C, D and E is p<0.05, values based on Student's t test. All error bars correspond to ± s.e.m.
(8.92 MB TIF)
