# Supplementary figures and images for: Progression of Parkinson's Disease Pathology Is Reproduced by Intragastric Administration of Rotenone in Mice
Source: PLoS One. 2010 Jan 19;5(1):e8762. doi: 10.1371/journal.pone.0008762 (PMC2808242; doi:10.1371/journal.pone.0008762)

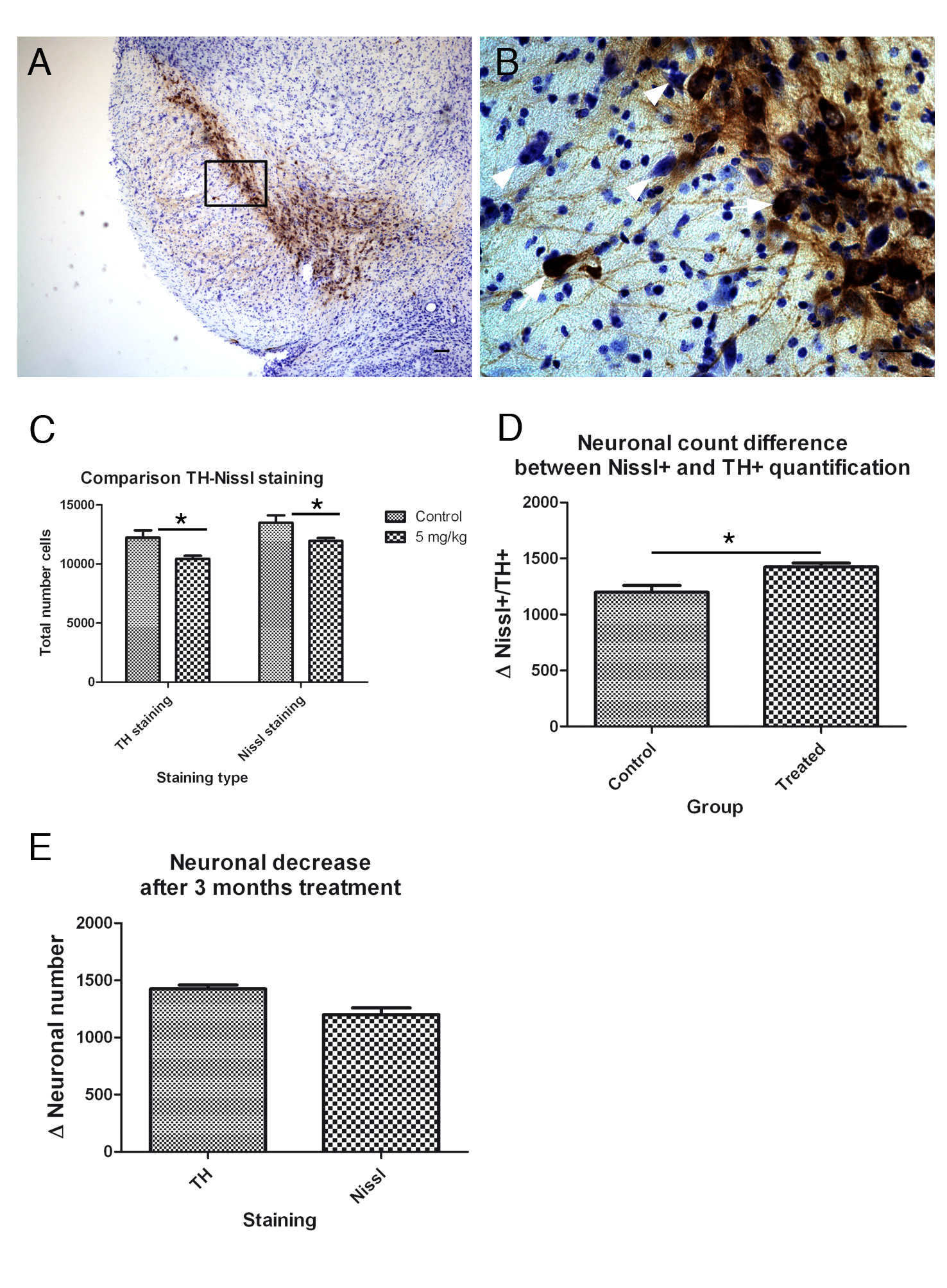

Supplement: Figure S3 — Rotenone treatment causes neuronal depletion and TH down-regulation in the SNc. (A, scale bar 80 µm, B, scale bar 20 µm) A, B, DAB against TH stained midbrain sections counterstained using Nissl's staining from 3 month treated mice. Arrowheads in B are Nissl+ but not TH+ neurons, whereas arrows in B are Nissl+−TH+ neurons. C, stereological quantification of total TH+ and total Nissl+ neurons in the SNc of 3 month control and treated mice. D, difference in the total number of cells between stereological quantification based on Nissl's and TH stainings in 3 month control and treated mice. E, neuronal decrease using DAB against TH (left column) and Nissl stainings (right column). * in C, D and E is p<0.05, values based on Student's t test. All error bars correspond to ± s.e.m. (8.92 MB TIF) [file pone.0008762.s003.tif]
